# Factors associated with family caregivers’ attitudes toward deprescribing in long-term care facilities: a cross-sectional study in South Korea

**DOI:** 10.1186/s12877-026-08333-6

**Published:** 2026-10-01

**Authors:** Ji-Hye Jung, Woo-young Shin, Jung-Ha Kim

**Affiliations:** 1 Department of Family Medicine, Chung-Ang University College of Medicine, 102 Heukseok-ro, Dongjak-gu, Seoul, 06973 Republic of Korea; 2 Department of Family Medicine, Chung-Ang University Gwangmyeong Hospital, Chung-Ang University College of Medicine, 102 Heukseok-ro, Dongjak-gu, Seoul, 06973 Republic of Korea

**Keywords:** Deprescribing, Long-term care facilities, Family caregivers, Polypharmacy, Attitude, K-rPATD

## Abstract

**Background:**

Deprescribing has emerged as an important strategy for addressing polypharmacy among older adults in long-term care facilities (LTCFs). Family caregivers often play a key role in medication-related decision-making, yet factors associated with caregivers’ attitudes toward deprescribing remain insufficiently understood. This study aimed to identify the factors associated with caregivers’ attitudes toward deprescribing among residents of LTCFs in South Korea.

**Methods:**

A cross-sectional online survey was conducted with 973 family caregivers of residents in LTCFs. Attitudes toward deprescribing were assessed using the Korean version of the revised Patients’ Attitudes Towards Deprescribing questionnaire (K-rPATD). Multiple linear regression models examined the associations of 16 measured predictors, represented by 18 regression terms, with four K-rPATD subscale scores and two global items, entered simultaneously. Unadjusted and Benjamini-Hochberg false discovery rate (FDR)-adjusted p values were computed for all 108 coefficients.

**Results:**

Of the 973 caregivers included, 56.0% were male, 88.0% had a college education or higher, and 73.0% reported dual-track prescriptions. Among the K-rPATD subscales, involvement showed the highest mean score (3.63 ± 0.61), followed by burden (3.61 ± 0.72) and concerns about stopping (3.33 ± 0.64), whereas the reverse-scored appropriateness subscale had the lowest mean (2.52 ± 0.68). Residents took an average of 5.6 ± 3.5 medications, and 16.3% had experienced adverse drug events. After FDR correction, eight associations remained significant: adverse drug events with higher burden and concerns about stopping and lower appropriateness; medication cost with higher burden and concerns about stopping; number of medications with higher burden; and number of chronic conditions with higher concerns about stopping and involvement. All other nominally significant associations did not survive FDR correction and are considered exploratory.

**Conclusions:**

After correction for multiple comparisons, family caregivers’ attitudes toward deprescribing were associated with the resident’s medication-related circumstances — adverse drug events, medication cost, number of medications, and number of chronic conditions — rather than with caregivers’ own sociodemographic characteristics. These cross-sectional findings are hypothesis-generating, but they help identify the family caregivers with whom deprescribing is most likely to be a relevant topic of discussion.

**Supplementary Information:**

The online version contains supplementary material available at https://doi.org/10.1186/s12877-026-08333-6.

## Background

 Population aging and the increasing prevalence of multimorbidity have made polypharmacy a major challenge in the care of older adults worldwide [1, 2]. Polypharmacy is commonly defined as the concurrent use of five or more medications and has been associated with an increased risk of adverse drug events (ADEs), falls, cognitive impairment, hospitalization, and mortality [3]. Its prevalence also rises steeply over the last year of life, when the balance of benefit and harm is least favorable [4]. Older adults residing in long-term care facilities (LTCFs) are particularly vulnerable to medication-related harm and ADEs owing to functional frailty and multiple chronic conditions. Polypharmacy is highly prevalent in this setting, with a systematic review reporting that up to 91% of LTCF residents take five or more medications [5], and the use of potentially inappropriate medications (PIMs) among older adults has been associated with an increased risk of hospitalization [6]. In South Korea, residents of LTCFs have been reported to take an average of 10.6 medications, with 45.7% taking 11 or more, and PIM use has been reported in approximately 41% [7].

Deprescribing is defined as the systematic process of dose reduction or discontinuation of medications that may no longer be appropriate or whose potential harm outweighs the benefits in the context of a patient’s health status and treatment goals [8, 9]. Deprescribing has emerged as a key strategy for addressing polypharmacy. Evidence suggests that deprescribing is a feasible intervention and that, when medications are reviewed against prespecified criteria, it can reduce mortality and falls among nursing home residents [10, 11]. However, the implementation of deprescribing in routine clinical care remains limited, both internationally and in South Korea.

Deprescribing is a complex process influenced by multidimensional factors, including physicians’ prescribing routines and fragmented healthcare environments in which medications are prescribed by multiple institutions [12]; in LTCFs these are compounded by facility-level demands. The Knowledge-Attitude-Practice (KAP) framework, which relates what individuals know and believe to what they actually do [13], places attitudes between knowledge and practice and provides a useful lens for understanding this process. Older adults with cognitive impairment depend more heavily on family and professional caregivers to manage their medicines [14], and in LTCF settings, where many residents have cognitive impairment and limited health literacy, family caregivers often act as surrogate decision-makers. Consequently, successful deprescribing requires more than the clinical decisions of healthcare professionals; it depends heavily on caregivers’ attitudes and perceptions toward reducing medications [15, 16]. Specifically, the multidimensional attitudes of patients and their caregivers, including perceived medication burden, perceptions of medication appropriateness, concerns about stopping medications, and willingness to participate in decision-making, can significantly influence the acceptance and implementation of deprescribing [17, 18].

To systematically evaluate these multidimensional attitudes, the revised Patients’ Attitudes Towards Deprescribing (rPATD) questionnaire has been widely used [19]. Previous quantitative studies of family caregivers’ attitudes toward deprescribing have been conducted mainly in community, hospital, or mixed care settings and have largely focused on willingness to deprescribe as a single outcome [16, 20, 21]. In addition, a growing body of qualitative research has explored patients’, caregivers’, and clinicians’ perspectives on deprescribing, including in long-term care settings [12, 22, 23]. However, few studies have examined the broader range of caregiver-, resident-, and medication-related factors associated with caregivers’ attitudes toward deprescribing in LTCF settings. Therefore, this study aimed to investigate the factors associated with attitudes toward deprescribing among family caregivers of older adults residing in LTCFs using the Korean version of the rPATD questionnaire (K-rPATD). Specifically, this study examined a broad range of caregiver-, resident-, and medication-related factors. By identifying these associations, this study aimed to provide empirical evidence for the development of tailored deprescribing strategies optimized for the LTCF environment.

## Methods

### Study design and participants

This cross-sectional survey was conducted among family caregivers of older adults residing in LTCFs in South Korea.

In this study, participants were family caregivers — that is, unpaid caregivers of a resident currently living in an LTCF. This corresponds to the criterion used for the caregivers’ version of the rPATD [19]: any person with a role in a family member’s or friend’s management of health and/or medications. Eligibility was determined by a single mandatory screening question at the start of the survey, which asked whether the respondent was a caregiver of an LTCF resident; respondents answering “no” were automatically exited from the survey, and no further eligibility criterion was applied. The Korean term used in this item denotes a family caregiver and is lexically distinct from the term for a paid care worker; facility staff and paid care workers were therefore not eligible. For brevity, family caregivers are referred to hereafter as caregivers. Within this group, respondents were additionally asked whether they were the resident’s “primary caregiver” — defined in the questionnaire as the caregiver most knowledgeable about the resident’s medical history, medication use, and functional status, irrespective of who held legal guardianship or acted as the family’s representative. This was recorded as a descriptive characteristic and was not used to determine eligibility. The respondent’s relationship to the resident was recorded separately using eight response options, grouped for analysis into children (including sons- and daughters-in-law), grandchildren, spouse, and others (siblings, parents, other relatives, and friends or neighbors; Table 1). Almost all respondents were relatives of the resident; six (0.6%) were friends or neighbors. Where a respondent cared for more than one LTCF resident, they were instructed to answer with respect to the resident whose condition they knew best. Caregiver status and all resident-related information—including medical history, chronic conditions, functional status (long-term care grade), prescribing pathway, and medication use—were self-reported and were not independently verified against medical or pharmacy records.

**Table 1:** General characteristics of family caregivers and long-term care facility residents (*N* = 973)

Variable	Category	*n* (%) or mean ± SD
[Family caregiver characteristics]		
Sex	Male	545 (56.0)
	Female	428 (44.0)
Age group (years)	≤ 39	174 (17.9)
	40–49	337 (34.6)
	50–59	299 (30.7)
	60–69	130 (13.4)
	≥ 70	33 (3.4)
Education level	Middle school or lower	11 (1.1)
	High school	106 (10.9)
	College or higher	856 (88.0)
Monthly household income (USD)ᵃ	< 1,449	43 (4.4)
	1,449–2,174	85 (8.7)
	2,175–2,899	179 (18.4)
	2,900–3,623	200 (20.6)
	≥ 3,624	466 (47.9)
Relationship to the resident	Children (including sons-in-law and daughters-in-law)	701 (72.0)
	Grandchildren	135 (13.9)
	Spouse	18 (1.8)
	Othersᵇ	119 (12.2)
Primary caregiver	Yes	841 (86.4)
	No	132 (13.6)
[Resident characteristics]		
Sex	Male	441 (45.3)
	Female	532 (54.7)
Age group (years)	≤ 69	79 (8.1)
	70–79	267 (27.4)
	80–89	454 (46.7)
	≥ 90	173 (17.8)
Duration of stay in the facility	< 1 year	119 (12.2)
	1 to < 3 years	531 (54.6)
	3 to < 5 years	148 (15.2)
	≥ 5 years	175 (18.0)
Long-term care grade	Grade 1	70 (7.2)
	Grade 2	226 (23.2)
	Grade 3	291 (29.9)
	Grade 4	119 (12.2)
	≥Grade 5^c^	267 (27.4)
Dementia	Yes	464 (47.7)
	No	509 (52.3)
Number of chronic conditions^d^	Mean ± SD	2.2 ± 1.8

*SD* Standard deviation

ᵃ USD values were calculated using the average exchange rate during the survey period (July–September 2025; 1 USD ≈ 1,380 KRW)

ᵇ “Others” includes siblings, parents, other relatives, and friends or neighbors

ᶜ Includes residents assigned Grade 5 and those not assigned a grade under the long-term care insurance system because their functional status was milder than Grade 5

ᵈ Total number of physician-diagnosed chronic conditions (range, 0–18). The most common were dementia (47.7%), hypertension (28.4%), diabetes mellitus (24.3%), stroke (21.7%), and arthritis (14.4%)

Data from the 2023 Long-Term Care Insurance Statistical Yearbook were used to estimate the required sample size. As official statistics on the number of caregivers were not available, the number of facility-based long-term care beneficiaries (199,592 individuals) was used as a proxy for the total caregiver population (N). A minimum of 384 respondents was required to estimate a population proportion at a 95% confidence level with a 5% margin of error [24]. In the study protocol this minimum was doubled to allow for the inclusion of both primary and non-primary caregivers and increased by approximately 30% to allow for attrition, giving a target of 1,000 for the parent survey (2 × 384 × 1.3 ≈ 1,000); this target also satisfied the sample-size recommendations for the psychometric analyses reported separately [25]. This calculation was based on estimating a population proportion and does not represent a formal power calculation for the multivariable regression analyses reported here.

Data were collected between July and September 2025 via an online survey administered by a professional research agency using a nationwide general-population online panel. A total of 1,000 respondents aged 19 years or older who were caregivers of residents in LTCFs and voluntarily agreed to participate were enrolled in the survey. The 973 caregivers analyzed here are a subset of the 1,000 respondents included in our previously published study that validated the K-rPATD [25]; the 27 participants excluded below were retained in that validation analysis, which did not require prescribing-pathway information. The two studies also differ in purpose: the earlier study established the psychometric properties (factor structure and reliability) of the instrument, whereas the present study uses the validated instrument to examine associations of caregiver-, resident-, and medication-related factors with attitudes toward deprescribing. The factor structure was not re-estimated here, and the participant characteristics in Table 1 and the item-level scores in Table 3 therefore partly overlap with those reported previously. Among these respondents, 27 were excluded: 26 who could not identify the resident’s prescribing pathway and one who did not respond to the question regarding external medication procurement. In total, 973 participants were included in the final analysis (Table 1). The research agency did not provide the number of panel members invited or screened out before enrolment, so a participation rate could not be calculated. Excluded and retained respondents did not differ significantly in caregiver sex, age, education, or income, or in resident sex, number of medications, or ADE experience (all *p* > 0.05); the excluded group included a lower proportion of primary caregivers (44.4% vs. 86.4%), consistent with their more limited knowledge of the resident’s prescribing arrangements.

No missing values were identified for the main variables in the final dataset. The study protocol was approved by the Institutional Review Board of Chung-Ang University (IRB No. 1041078-20241129-HR-339), and informed consent was obtained electronically from all participants prior to participation.

### Measures

The survey consisted of two main domains: the dependent variable (caregivers’ attitudes toward deprescribing) and independent variables encompassing caregiver characteristics, resident characteristics, and medication-related factors.

#### Dependent variable: caregivers’ attitudes toward deprescribing

Caregivers’ attitudes toward deprescribing were assessed using the K-rPATD. The K-rPATD consists of 19 items: 17 items across four factor domains (burden [four items], appropriateness [five items], concerns about stopping [four items], and involvement [four items]) and two global items assessing willingness to stop medications and overall satisfaction with current medications. All items were measured using a 5-point Likert scale ranging from 1 (strongly disagree) to 5 (strongly agree). Following the scoring convention adopted in the validation study [25], higher factor scores indicate greater perceived medication burden, greater concerns about stopping, and greater involvement in medication decision-making; the appropriateness factor is reverse-scored, so that a higher score indicates a stronger belief that the resident’s current medicines are appropriate. Item-level means in Table 3 are presented in the original response direction, in which higher values denote stronger agreement with the item as worded. For descriptive purposes, the agreement rate for each item was defined as the proportion of respondents who answered “agree (4)” or “strongly agree (5).”

#### Independent variables

##### Caregiver and resident characteristics

Caregiver variables included sex, age group, education level, monthly household income, and relationship to the resident. Resident variables included sex, age group, duration of stay in the facility, long-term care grade, presence of dementia, and total number of chronic conditions.

Long-term care grade is a standardized classification under the Korean National Long-Term Care Insurance System that reflects an older adult’s level of functional dependency and care needs. Grades range from 1 to 5, with Grade 1 indicating the most severe functional impairment and the highest level of care need and Grade 5 indicating relatively mild impairment; thus, a higher grade number corresponds to a lower level of functional dependency. Residents whose functional status was milder than Grade 5 were not assigned a grade because this represented the mildest end of the functional dependency spectrum and were combined with Grade 5 into a single category (Grade 5 or above).

##### Medication-related factors

Five medication-related factors were assessed as independent variables: prescribing pathway type, number of medications, monthly medication costs, ADEs, and monthly dietary supplement costs. Prescribing pathway type was determined by the source of the residents’ prescriptions. Facility-only prescribing was defined as a model in which all residents’ medications were prescribed solely by the physician responsible for the LTCF, typically a contracted physician providing periodic on-site visits. Dual-track prescribing was defined as a model in which the resident received prescriptions from the facility physician and one or more external healthcare providers (i.e., outpatient clinics or hospitals located outside the facility). The number of medications was defined as the total number of medications that the resident was currently taking. Monthly medication cost was assessed using five response categories: <10,000 KRW (< 7.2 USD), 10,000–49,999 KRW (7.2–36.2 USD), 50,000–99,999 KRW (36.2–72.5 USD), 100,000–149,999 KRW (72.5–108.7 USD), and ≥ 150,000 KRW (≥ 108.7 USD). South Korean won (KRW) was converted to US dollars (USD) using the average exchange rate during the survey period (1 USD = 1,380 KRW; July–September 2025) [26]. Experience of ADEs was assessed by asking whether the resident had experienced any ADE within the past 3 months (yes, no, or unknown). In this study, an ADE was defined as an adverse effect experienced by the resident while taking medications. Monthly dietary supplement costs were assessed using the same five response categories as monthly medication costs.

### Statistical analysis

Descriptive statistics, including frequencies, percentages, means, and standard deviations, were used to summarize participants’ general characteristics and medication management status. The psychometric properties of the K-rPATD were evaluated previously in the same survey [25]: overall internal consistency was high (Cronbach’s alpha 0.881) and was acceptable for the burden (0.781) and appropriateness (0.766) subscales, but lower for the involvement (0.637) and concerns about stopping (0.563) subscales, and the four-factor model showed marginal fit. Findings for the latter two domains should therefore be interpreted with corresponding caution. Multiple linear regression analyses were conducted to identify factors associated with the K-rPATD subscales and global items.

The final models comprised caregiver characteristics (sex, age group, education level, monthly household income, and relationship to the resident), resident characteristics (sex, age group, duration of facility stay, long-term care grade, presence of dementia, and total number of chronic conditions), and five medication-related factors. All variables were entered simultaneously and were mutually adjusted. The number of medications and chronic conditions were entered as continuous variables. Age group, education level, monthly household income, duration of stay, long-term care grade, monthly medication cost, and monthly dietary supplement cost were modeled as ordinal variables, assuming a linear trend across categories. Because the category widths of these variables are unequal, the linear specification was tested formally: for each ordinal predictor and each outcome, a model treating the variable as an unordered categorical term was compared with the corresponding linear model using an F test for departure from linearity. No departure from linearity was detected for monthly medication cost in any domain (all *p* ≥ 0.46) or for any other ordinal predictor (all *p* ≥ 0.06), with the single exception of dietary supplement cost in the satisfaction model (*p* = 0.033). That predictor was therefore also modeled categorically for that outcome: the categorical term was jointly associated with satisfaction (F(4, 951) = 2.74, *p* = 0.027) in a non-monotonic pattern, while the remaining coefficients were essentially unchanged. Because this association was nominal and would not survive correction for multiple comparisons, and because retaining an identical specification in all six models keeps the coefficients comparable across domains, the linear term was retained in the main models and the categorical result is reported as a sensitivity analysis. Categorical estimates for monthly medication cost and for dietary supplement cost are reported in Supplementary Table S2. Each ordinal coefficient is therefore interpreted as the average change in the outcome per one-category increase. Sex, presence of dementia, prescribing pathway type (dual-track vs. facility-only), and experience of ADEs (yes vs. no/unknown) were entered as binary variables. Relationship to the resident was entered as three dummy variables (spouse, grandchild, and other), with children as the reference group; all categories were retained in every model and are reported for all outcomes in Table 4. All VIFs were below 2.1 (maximum 2.02, mean 1.29), indicating no meaningful multicollinearity. Additional regression diagnostics were assessed, including linearity, homoscedasticity (Breusch-Pagan test), residual distribution, and influential observations (Cook’s distance). Homoscedasticity was satisfied in five of the six models, with a marginal violation in the appropriateness model (*p* = 0.03), and residuals showed mild departures from normality consistent with Likert-derived subscale scores; given the large sample (*n* = 973) and the HC3 sensitivity analyses, these were unlikely to materially affect inference. Linearity was assessed by the F tests for departure from linearity described above and by inspection of residual-versus-fitted plots, which showed no systematic curvature. No observation was influential: the maximum Cook’s distance was 0.029 across all six models, and no observation exceeded the conventional threshold of 1. As a sensitivity analysis, all models were refitted with heteroscedasticity-consistent (HC3) standard errors. Because each of the six outcomes was modeled with 16 measured predictors represented by 18 regression terms (relationship to the resident contributing three dummy variables), yielding 108 coefficients in total, we distinguished robust associations from weaker findings that could reflect chance: unadjusted, Benjamini-Hochberg false discovery rate (FDR)-adjusted, and HC3-robust p values for every coefficient are provided in Supplementary Table S1, and associations that did not survive FDR adjustment are interpreted as exploratory throughout. Because “unknown” responses on the ADE item represented a substantial subgroup, the yes-versus-no/unknown coding was examined in a sensitivity analysis that modeled ADE experience as a three-level variable (reference = no) and, separately, that excluded “unknown” responses. The principal ADE associations with burden, appropriateness and concerns about stopping were unchanged in both analyses, whereas the weaker association with willingness to stop medications was attenuated and no longer nominally significant when “unknown” responses were excluded (B = 0.146, *p* = 0.069). Because willingness to stop medications and satisfaction are single Likert items rather than multi-item means, these two models were additionally refitted using ordinal logistic regression; the direction of association agreed with the linear models for 35 of the 36 coefficients (the exception being a coefficient indistinguishable from the null). Nominal significance differed for the association between the number of chronic conditions and willingness to stop medications, but no FDR-based or principal conclusion changed (Supplementary Table S3). Finally, adding primary-caregiver status as a covariate did not materially alter the principal associations (Supplementary Table S3).

The regression results were presented as unstandardized regression coefficients (B) with 95% confidence intervals (CIs). All analyses were performed in R version 4.6.0 (R Foundation for Statistical Computing, Vienna, Austria), using the car (variance inflation factors), sandwich and lmtest (HC3 robust standard errors), and base stats (Benjamini-Hochberg FDR adjustment) packages. Statistical significance was set at *p* < 0.05, and unadjusted, FDR-adjusted and HC3-robust p values are reported for all coefficients.

## Results

### General characteristics of the study participants

The general characteristics of the 973 participants are presented in Table 1.

Among family caregivers, 56.0% were male and 44.0% were female. The most common age groups were individuals in their 40s (34.6%) and 50s (30.7%). A large proportion of caregivers had a college education or higher (88.0%), and 47.9% reported a monthly household income of 5 million KRW (approximately 3,620 USD) or more. Regarding the relationship to the resident, children (including daughters-in-law and sons-in-law) accounted for the largest proportion (72.0%). Furthermore, 86.4% of respondents identified themselves as the primary caregivers of residents.

Among residents of LTCFs, 54.7% were female and 45.3% were male. The largest proportion of residents were in their 80s (46.7%). The most common length of stay in the facility was 1 to 3 years (54.6%). Long-term care Grade 3 was the most frequently reported (29.9%). Dementia was present in 47.7% of residents, and the mean number of chronic conditions was 2.2 ± 1.8. The most commonly reported conditions included dementia (47.7%), hypertension (28.4%), diabetes mellitus (24.3%), stroke (21.7%), and arthritis (14.4%).

### Caregiver-reported medication use and medication management characteristics of LTCF residents

The medication management and prescribing patterns of residents are presented in Table 2.

**Table 2:** Medication-related factors among long-term care facility residents (*N* = 973)

Variable	Category	*n* (%) or Mean ± SD
Number of medications	Mean ± SD	5.6 ± 3.5
Experience of adverse drug events within the past 3 months	Yes	159 (16.3)
	No	629 (64.6)
	Unknown	185 (19.0)
Prescribing pathway type	Dual-track (facility and external providers)	710 (73.0)
	Facility-only	263 (27.0)
Monthly medication cost (USD)^a^	I (< 7.2)	67 (6.9)
	II (7.2–36.2)	250 (25.7)
	III (36.2–72.5)	294 (30.2)
	IV (72.5–108.7)	228 (23.4)
	V (≥ 108.7)	134 (13.7)
Monthly dietary supplement cost (USD)	I (< 7.2)	180 (18.5)
	II (7.2–36.2)	238 (24.5)
	III (36.2–72.5)	292 (30.0)
	IV (72.5–108.7)	175 (18.0)
	V (≥ 108.7)	88 (9.0)

*SD* Standard deviation

^a^ USD values were calculated using the average exchange rate during the survey period (July–September 2025; 1 USD ≈ 1,380 KRW)

Residents took an average of 5.6 ± 3.5 medications. During the previous 3 months, 16.3% of residents had experienced ADEs. Regarding prescription pathways, 73.0% of caregivers reported that residents received facility-based prescriptions and prescriptions from external healthcare providers, compared with 27.0% who reported facility-based prescriptions only. The monthly medication cost was most commonly in the range of 50,000–99,999 KRW (36.2–72.5 USD; 30.2%), and the monthly dietary supplement cost was most commonly 50,000–99,999 KRW (36.2–72.5 USD; 30.0%). Among caregivers reporting the use of external prescriptions, the most frequently reported reasons were trust in external healthcare institutions or physicians (44.5%) and continuity of long-term prescriptions for the same condition (43.4%).

### Caregivers’ attitudes toward deprescribing (K-rPATD scores)

The subscale scores, item-level results, and agreement rates for the K-rPATD are presented in Table 3.

**Table 3:** Family caregivers' attitudes toward deprescribing (K-rPATD) (*N* = 973). Score range: 1–5 (1 = Strongly disagree; 5 = Strongly agree)

Factor and item	Mean ± SD	Agreement, n (%)^a^
Burden	3.61 ± 0.72	
My care recipient's medicines are quite expensive.	3.52 ± 0.87	543 (55.8)
I feel that the person I care for is taking a large number of medicines.	3.72 ± 0.92	622 (63.9)
I feel that my care recipient's medicines are a burden to them.	3.42 ± 0.97	478 (49.1)
Sometimes I think the person I care for takes too many medicines.	3.78 ± 0.97	632 (65.0)
Appropriateness	2.52 ± 0.68	
I feel that the person that I care for may be taking one or more medicines that they no longer need.	3.70 ± 0.89	608 (62.5)
I would like the doctor to try stopping one of my care recipient's medicines to see how they feel without it.	3.46 ± 0.97	512 (52.6)
I would like the doctor to reduce the dose of one or more of my care recipient's medicines.	3.23 ± 0.99	377 (38.7)
I think one or more of my care recipient's medicines may not be working.	3.65 ± 0.91	582 (59.8)
I believe one or more of my care recipient's medicines may be currently giving them side effects.	3.38 ± 0.96	456 (46.9)
Concerns about stopping	3.33 ± 0.64	
I would be reluctant to stop one of my care recipient's medicines that they had been taking for a long time.	3.20 ± 1.04	401 (41.2)
I get stressed whenever changes are made to my care recipient's medicines.	3.05 ± 1.04	340 (34.9)
I feel that if I agreed to stopping one of my care recipient's medicines then this is giving up on them.	3.32 ± 0.87	415 (42.7)
The person that I care for has had a bad experience when stopping a medicine before.	3.77 ± 0.90	638 (65.6)
Involvement	3.63 ± 0.61	
I know exactly what medicines the person that I care for is currently taking and/or I have an up-to-date list of their medicines.	3.77 ± 0.89	645 (66.3)
I like to know as much as possible about my care recipient's medicines.	3.67 ± 0.86	592 (60.8)
I like to be involved in making decisions about my care recipient's medicines with their doctors.	3.72 ± 0.90	618 (63.5)
I always ask the doctor, pharmacist, or other healthcare professional if there is something I don't understand about my care recipient's medicines.	3.35 ± 0.85	400 (41.1)
Global items		
If their doctor said it was possible I would be willing to stop one or more of my care recipient's medicines.	3.65 ± 0.87	606 (62.3)
Overall, I am satisfied with my care recipient's current medicines.	3.33 ± 0.81	394 (40.5)

*K-rPATD* Korean version of the revised Patients' Attitudes Towards Deprescribing questionnaire, *SD* Standard deviation

^a^ Agreement was defined as a response of "agree (4)" or "strongly agree (5)." The appropriateness factor is reverse-scored, so that a higher factor score indicates a stronger belief that the resident's current medicines are appropriate; item-level means are shown in the original response direction, in which higher values denote stronger agreement with the item as worded

Among the four subscales, involvement showed the highest mean score (3.63 ± 0.61), followed by burden (3.61 ± 0.72) and concerns about stopping (3.33 ± 0.64), whereas the reverse-scored appropriateness subscale had the lowest mean (2.52 ± 0.68), indicating that caregivers on average did not regard the resident’s current medicines as fully appropriate. For the global items, willingness to stop medications if recommended by a physician (3.65 ± 0.87) was higher than satisfaction with current medications (3.33 ± 0.81).

At the item level, the highest mean scores were observed for awareness of taking too many medications (item 4: 3.78 ± 0.97), previous negative experiences when stopping medications (item 13: 3.77 ± 0.90), and awareness of the current medication list (item 14: 3.77 ± 0.89). In contrast, stress associated with changes in prescriptions (item 11: 3.05 ± 1.04) showed the lowest mean score.

Regarding agreement rates (defined as the proportion of respondents selecting “agree” or “strongly agree”), the highest agreement rates were observed for awareness of the current medication list (item 14: 66.3%), previous negative experiences when stopping medications (item 13: 65.6%), and awareness of taking too many medications (item 4: 65.0%). Conversely, the lowest agreement rates were observed for stress associated with prescription changes (item 11: 34.9%), desire to reduce medication doses (item 7: 38.7%), and overall satisfaction with current medications (item 19: 40.5%), indicating that fewer than half of the caregivers reported satisfaction with the residents’ current medications. For the global items, 62.3% of caregivers agreed that they would be willing to stop one or more of the resident’s medications if their physician said it was possible.

### Factors associated with K-rPATD scores

The results of the multiple linear regression analyses examining factors associated with the K-rPATD subscales and global items are presented in Table 4. All variables, including caregiver characteristics, resident characteristics, and medication-related factors, were entered simultaneously and were mutually adjusted.

**Table 4:** Factors associated with family caregivers’ attitudes toward deprescribing (K-rPATD): multiple linear regression analysis (*N* = 973)

Independent variables	Burden B (95% CI)	Appropriateness B (95% CI)	Concerns about stopping B (95% CI)	Involvement B (95% CI)	Willingness to stop medications B (95% CI)	Satisfaction B (95% CI)
[Family caregiver characteristics]						
Sex (female vs. male)	-0.005 (-0.098, 0.089)	-0.047 (-0.136, 0.041)	-0.044 (-0.126, 0.037)	-0.047 (-0.126, 0.033)	-0.074 (-0.189, 0.041)	-0.110 (-0.217, -0.003)
Age group (per 1-category increase)	0.013 (-0.047, 0.074)	-0.014 (-0.071, 0.044)	0.010 (-0.042, 0.063)	-0.027 (-0.079, 0.024)	-0.032 (-0.106, 0.042)	-0.020 (-0.089, 0.049)
Education level (per 1-level increase)	0.022 (-0.109, 0.154)	0.001 (-0.123, 0.126)	-0.102 (-0.217, 0.012)	-0.019 (-0.131, 0.094)	0.037 (-0.125, 0.199)	-0.154 (-0.305, -0.003)
Monthly household income (per 1-category increase)	0.021 (-0.020, 0.062)	-0.023 (-0.062, 0.015)	0.020 (-0.015, 0.056)	0.036 (0.001, 0.071)	0.046 (-0.004, 0.096)	0.048 (0.002, 0.095)
Relationship to the resident: grandchild (vs. children)	-0.008 (-0.177, 0.161)	0.026 (-0.133, 0.186)	-0.007 (-0.154, 0.140)	-0.022 (-0.166, 0.121)	-0.062 (-0.269, 0.145)	0.026 (-0.167, 0.219)
Relationship to the resident: spouse (vs. children)	-0.276 (-0.632, 0.081)	0.010 (-0.327, 0.347)	0.055 (-0.256, 0.365)	-0.154 (-0.458, 0.150)	-0.154 (-0.591, 0.284)	-0.264 (-0.671, 0.144)
Relationship to the resident: other (vs. children)	0.029 (-0.111, 0.168)	-0.055 (-0.187, 0.077)	0.078 (-0.044, 0.200)	-0.036 (-0.156, 0.083)	-0.074 (-0.246, 0.098)	-0.227 (-0.387, -0.067)
[Resident characteristics]						
Sex (female vs. male)	-0.024 (-0.120, 0.072)	0.046 (-0.045, 0.137)	-0.064 (-0.148, 0.019)	0.013 (-0.069, 0.095)	0.103 (-0.015, 0.221)	0.013 (-0.097, 0.123)
Age group (per 1-category increase)	0.006 (-0.063, 0.074)	-0.031 (-0.095, 0.034)	-0.014 (-0.073, 0.046)	0.023 (-0.035, 0.082)	0.102 (0.018, 0.185)	-0.045 (-0.123, 0.033)
Duration of stay (per 1-category increase)	0.005 (-0.045, 0.056)	0.001 (-0.047, 0.049)	0.011 (-0.033, 0.055)	-0.011 (-0.054, 0.033)	-0.039 (-0.101, 0.023)	0.000 (-0.058, 0.058)
Long-term care grade (per 1-grade increase)	0.009 (-0.027, 0.045)	0.000 (-0.034, 0.034)	-0.022 (-0.054, 0.009)	-0.017 (-0.048, 0.014)	0.010 (-0.034, 0.054)	-0.028 (-0.069, 0.014)
Dementia (yes vs. no)	0.010 (-0.082, 0.103)	-0.037 (-0.125, 0.050)	-0.004 (-0.085, 0.076)	-0.031 (-0.110, 0.048)	-0.014 (-0.128, 0.100)	-0.067 (-0.173, 0.039)
Number of chronic conditions (per 1-condition increase)	0.012 (-0.014, 0.039)	-0.015 (-0.040, 0.010)	0.038 (0.015, 0.062)	0.038 (0.015, 0.060)	0.030 (-0.003, 0.063)	0.035 (0.005, 0.065)
[Medication-related factors]						
Number of medications (per 1-medication increase)	0.026 (0.013, 0.039)	-0.015 (-0.027, -0.002)	-0.007 (-0.018, 0.005)	0.006 (-0.006, 0.017)	0.012 (-0.004, 0.028)	-0.006 (-0.021, 0.009)
Experience of adverse drug events (yes vs. no/unknown)	0.206 (0.080, 0.333)	-0.208 (-0.327, -0.088)	0.255 (0.146, 0.365)	0.068 (-0.039, 0.176)	0.166 (0.011, 0.321)	-0.061 (-0.205, 0.083)
Dual-track prescribing (vs. facility-only)	0.064 (-0.042, 0.170)	-0.079 (-0.179, 0.021)	0.108 (0.016, 0.200)	0.052 (-0.039, 0.142)	0.085 (-0.045, 0.215)	-0.055 (-0.176, 0.067)
Monthly medication cost (per 1-tier increase)	0.085 (0.038, 0.133)	-0.057 (-0.101, -0.012)	0.067 (0.026, 0.109)	0.026 (-0.014, 0.066)	0.055 (-0.003, 0.113)	-0.048 (-0.103, 0.006)
Monthly dietary supplement cost (per 1-tier increase)	-0.008 (-0.052, 0.036)	-0.021 (-0.063, 0.021)	0.002 (-0.037, 0.040)	0.003 (-0.035, 0.040)	-0.017 (-0.071, 0.037)	0.038 (-0.013, 0.088)
R² (adjusted R²)	0.072 (0.055)	0.063 (0.046)	0.098 (0.081)	0.044 (0.026)	0.045 (0.027)	0.033 (0.015)

*B* Unstandardized regression coefficient, *CI* Confidence interval, *K-rPATD* Korean version of the revised Patients’ Attitudes Towards Deprescribing questionnaire

All variables were entered simultaneously and mutually adjusted. Education level and monthly household income were modeled as ordinal variables. Number of medications and number of chronic conditions were entered as continuous variables. Age group, duration of stay, long-term care grade, monthly medication cost, and monthly dietary supplement cost were modeled as ordinal variables. Sex, dementia, prescribing pathway, and adverse drug events were entered as binary variables, whereas relationship to the resident was entered using dummy variables, with children as the reference group. All independent variables are shown in this table

Variables are presented in the same order as in Tables 1 and 2. Coefficients are presented as B (95% CI) for all variables. Exclusion of zero from the 95% CI indicates nominal, unadjusted significance; exact, false discovery rate (FDR)-adjusted, and heteroscedasticity-consistent (HC3) p values for all coefficients are reported in Supplementary Table S1, and associations with an FDR-adjusted *p* ≥ 0.05 are interpreted as exploratory. The appropriateness factor is reverse-scored, so that a positive coefficient indicates a stronger belief that current medicines are appropriate. A higher long-term care grade number indicates milder functional impairment

At the nominal, unadjusted *p* < 0.05 level, the following associations were observed. Among caregiver characteristics, female sex (B = − 0.110, 95% CI: −0.217 to − 0.003, *p* = 0.044) and higher education level (B = − 0.154, 95% CI: −0.305 to − 0.003, *p* = 0.046) were significantly associated with lower satisfaction with current medications. Caregivers in the “others” relationship category also reported significantly lower satisfaction than children (B = − 0.227, 95% CI: −0.387 to − 0.067, *p* = 0.005). In contrast, higher monthly household income was associated with greater involvement (B = 0.036, 95% CI: 0.001–0.071, *p* = 0.043) and higher satisfaction (B = 0.048, 95% CI: 0.002–0.095, *p* = 0.042).

Among resident characteristics, older age was significantly associated with greater willingness to stop medications (B = 0.102, 95% CI: 0.018–0.185, *p* = 0.018). The number of chronic conditions was associated with three domains; each additional chronic condition was associated with higher scores for concerns about stopping (B = 0.038, 95% CI: 0.015–0.062, *p* = 0.001), involvement (B = 0.038, 95% CI: 0.015–0.060, *p* = 0.001), and satisfaction (B = 0.035, 95% CI: 0.005–0.065, *p* = 0.024).

Among medication-related factors, experience of ADEs was associated with higher perceived burden (B = 0.206, 95% CI: 0.080–0.333, *p* = 0.001), lower perceived appropriateness of the current medicines (B = − 0.208, 95% CI: −0.327 to − 0.088, *p* < 0.001), greater concerns about stopping (B = 0.255, 95% CI: 0.146–0.365, *p* < 0.001), and greater willingness to stop medications (B = 0.166, 95% CI: 0.011–0.321, *p* = 0.035). Each additional medication was associated with higher perceived burden (B = 0.026, 95% CI: 0.013–0.039, *p* < 0.001) and with lower perceived appropriateness (B = − 0.015, 95% CI: −0.027 to − 0.002, *p* = 0.023). Higher monthly medication cost was associated with greater burden (B = 0.085, 95% CI: 0.038–0.133, *p* < 0.001), lower perceived appropriateness (B = − 0.057, 95% CI: −0.101 to − 0.012, *p* = 0.013), and concerns about stopping (B = 0.067, 95% CI: 0.026–0.109, *p* = 0.001). Dual-track prescriptions were associated with greater concerns about stopping than facility-only prescriptions (B = 0.108, 95% CI: 0.016–0.200, *p* = 0.022). Monthly dietary supplement costs showed no evidence of a linear trend across cost tiers in any K-rPATD domain. In the satisfaction model, however, a departure from linearity was detected (*p* = 0.033), and when the variable was modeled categorically the term was jointly associated with satisfaction in a non-monotonic pattern (F(4, 951) = 2.74, *p* = 0.027); this association was nominal, would not survive FDR adjustment, and is therefore reported as exploratory (Supplementary Table S2).

Adjusted R² values ranged from 0.015 for satisfaction to 0.081 for concerns about stopping. After Benjamini-Hochberg FDR correction across all 108 coefficients, eight associations remained significant: adverse drug events with higher burden, lower perceived appropriateness, and higher concerns about stopping; monthly medication cost with burden and concerns about stopping; number of medications with burden; and number of chronic conditions with concerns about stopping and involvement. The remaining nominally significant associations, including adverse drug events with willingness to stop medications, monthly medication cost and number of medications with appropriateness (both in the direction of lower perceived appropriateness), number of chronic conditions with satisfaction, dual-track prescribing with concerns about stopping, and all associations involving caregiver and resident sociodemographic characteristics, did not survive FDR adjustment and are therefore interpreted as exploratory (Supplementary Table S1).

## Discussion

This study examined a broad range of caregiver-, resident-, and medication-related factors associated with family caregivers’ attitudes toward deprescribing among residents of LTCFs in South Korea. Among the factors examined, medication-related experiences, particularly ADEs and monthly medication costs, were the most consistent correlates of caregivers’ attitudes, being associated with greater perceived burden, greater concerns about stopping, and lower perceived appropriateness of current medicines, and dual-track prescriptions were associated with greater concerns about stopping medications, although this last association did not survive correction for multiple comparisons. Beyond these medication-related factors, the resident’s number of chronic conditions and the caregiver’s household income, education level, sex, and relationship to the resident were also associated with specific attitude domains, indicating that caregivers’ attitudes toward deprescribing are shaped by medication-related experiences and the broader caregiving context. Associations involving adverse drug events, medication cost, number of medications, and number of chronic conditions were the most robust, remaining significant after FDR correction, whereas those involving dual-track prescribing and caregiver and resident sociodemographic characteristics are discussed below as exploratory.

The number of medications was associated with higher perceived burden, an association that remained significant after FDR correction, and, as an exploratory finding, with lower perceived appropriateness of the current regimen; it was not associated with willingness to stop medications or concerns about stopping. This finding suggests that caregivers may recognize polypharmacy as problematic but may not consider deprescribing unless it is accompanied by tangible experiences, such as ADEs or financial burden [22]. Accordingly, education may be more effective when it is tailored to the individual’s situation and addresses the anticipated risks of continued use, rather than conveying general information alone [12]; patient-directed educational materials have been shown to increase engagement in deprescribing discussions [27]. This pattern was also reflected at the item level; although the majority of caregivers recognized excessive medication use (65.0% agreement), fewer than half expressed a desire to reduce medication doses (38.7%) or satisfaction with current medications (40.5%). This discrepancy between problem recognition and willingness to act suggests that awareness of polypharmacy alone may be insufficient to motivate deprescribing, reinforcing the need for targeted interventions that address this attitudinal gap. This pattern aligns with evidence that attitudinal factors, rather than external characteristics such as the number of medications, drive the desire to deprescribe: in a hospital-based study using the rPATD, a perceived absence of unnecessary medications and no desire for dose reduction were the strongest predictors of low desire to stop a medication, whereas age, comorbidity, and polypharmacy were poor predictors [21]. The generally favorable willingness observed in our sample (62.3% agreement) is also consistent with a meta-analysis reporting that approximately three-quarters of caregivers are willing to deprescribe their care recipients’ medications [16].

Monthly medication costs were associated with increased burden and concerns about stopping medication, both of which remained significant after FDR correction, and, as an exploratory association, with lower perceived appropriateness of the current medicines. These results suggest that economic burden may be associated with caregivers’ reconsideration of current medication regimens, although the cross-sectional design precludes any causal interpretation. In Korea, polypharmacy translates into substantially higher medication expenditure among older adults: in a national cohort study, annual drug costs were 536,721 KRW higher and total medical costs 872,018 KRW higher in those with polypharmacy than in those without [28]. Our measure captured the amount reported as spent each month on the resident’s prescribed medicines, and accumulated expenditure of this kind may be associated with greater acceptance of deprescribing; the survey did not, however, record what proportion of that cost was borne by the family. This is consistent with qualitative work identifying cost, alongside perceived effectiveness and adverse effects on quality of life, as a key determinant of the perceived value of a medication and of willingness to accept deprescribing [22]. Policy approaches similar to Japan’s medication adjustment incentives, whose introduction was followed by a 19.3% nationwide reduction in polypharmacy among adults aged 75–89 years, may provide a useful model for aligning patient safety with economic benefits [29].

Experience of ADEs was associated with the greatest number of K-rPATD domains; the associations with higher burden, lower perceived appropriateness, and higher concerns about stopping remained significant after FDR correction, whereas that with willingness to stop medications was exploratory. Caregivers who reported ADEs were more likely to question the appropriateness of current medications and recognize the need for deprescribing while simultaneously expressing greater concerns about stopping medications. This dual response highlights the complexity of caregivers’ decision-making in medication management and is consistent with qualitative findings that the experience of, or fear of, adverse outcomes shapes both the recognition of medication inappropriateness and apprehension about withdrawal [17, 30]. For caregivers who have experienced ADEs, clinicians may need to adopt shared decision-making approaches that include clear monitoring plans following deprescribing [12].

Another notable finding was that caregivers who received facility-based and external prescriptions reported greater concerns about stopping medications; this association was nominally significant but did not survive FDR correction and is therefore interpreted as exploratory. In Korean LTCFs, where physicians typically provide care through periodic visits rather than being continuously available on-site, caregivers often serve as intermediaries between multiple prescribers. Openness to deprescribing depends substantially on trust in the prescriber, which qualitative work has shown can act as either an enabler or a barrier [31]; an intermediary role distributed across several prescribers may weaken the continuity of relationship on which such trust is built. This fragmented communication structure may increase uncertainty regarding responsibility for medication decisions and contribute to caregivers’ concerns about deprescribing [7, 32]. Consistent with this observation, fragmented medical care has been identified as one of the main barriers to deprescribing in older adults with polypharmacy [33], and qualitative research has described how care fragmented across multiple prescribers, together with a lack of clinician continuity, impedes deprescribing [30]. Multidisciplinary medication review in care homes has been shown to improve the appropriateness of prescribing and to resolve medication-related problems, although its effect on resident-level outcomes remains uncertain and the supporting evidence is of low certainty [34]. Integrated medication information-sharing and multidisciplinary collaboration therefore warrant formal evaluation as approaches to these structural barriers.

Regarding resident characteristics, a greater number of chronic conditions was associated with higher scores for concerns about stopping and involvement, both of which remained significant after FDR correction, and, as an exploratory finding, with higher satisfaction. Caregivers of residents with greater multimorbidity may perceive each medication as being tied to the management of a specific condition, which could simultaneously heighten their engagement in medication-related decisions and their apprehension that discontinuing medications might destabilize disease control [17, 32]. Notably, it was the cumulative burden of chronic conditions, rather than the presence of dementia or the level of functional dependency, that was associated with caregivers’ attitudes, suggesting that these attitudes may be more closely linked to the perceived complexity of disease management than to residents’ overall clinical severity. In addition, older resident age was associated with greater willingness to stop medications, although this association did not survive FDR correction. This finding is consistent with previous reports that caregivers become more receptive to deprescribing as care goals shift toward comfort and quality of life at an advanced age [35]. In that study, family caregivers described prioritizing comfort over prolonging life and a general preference to limit medications, particularly those causing side effects or reducing comfort. This contrasts with a hospital-based study in which age was a poor predictor of the desire to deprescribe [21], suggesting that the association with older resident age may be specific to the LTCF context and its shift in care goals.

Caregiver characteristics were associated almost exclusively with satisfaction with current medications rather than with attitudes directly related to deprescribing. Female sex, higher education level, and an “other” relationship to the resident were associated with lower satisfaction, whereas higher household income was associated with greater satisfaction as well as greater involvement. Caregivers with higher education levels may have higher expectations regarding medication appropriateness and evaluate current regimens more critically, whereas caregivers with greater financial resources may have a greater capacity to engage actively in residents’ care. The lower satisfaction observed among caregivers other than children may reflect their more peripheral roles in care decision-making and limited access to medication-related information. However, given the modest effect sizes, these interpretations should be considered exploratory.

Although involvement showed the highest mean score among the K-rPATD subscales, none of the medication-related factors were significantly associated with this domain; only the number of chronic conditions, which remained significant after FDR correction, and household income, an exploratory association, showed significant associations. This suggests that caregivers’ engagement in medication decision-making may be driven more by their caregiving capacity and the complexity of the resident’s condition than by specific medication characteristics. Similarly, previous qualitative work reported that caregiver involvement in medication-related decisions is largely shaped by the broader care context and facility philosophy [23]. In addition, satisfaction with current medications was associated with caregivers’ sociodemographic background rather than with medication-related factors, supporting the view that satisfaction may reflect broader relational and contextual factors, such as trust in healthcare providers, rather than prescribing characteristics themselves. Family caregivers often report high levels of trust in clinicians and rely on their judgment when making deprescribing decisions [35].

This study has some limitations. First, because the study was cross-sectional and was designed to identify associations rather than causal relationships, the results should be interpreted as associations, and the direction of causality cannot be inferred. Second, participants were recruited from a general-population, reward-based online panel, and the resulting sample—predominantly male (56.0%), highly educated (88.0% with a college education or higher), relatively young, and containing very few spouse caregivers (1.8%)—is unlikely to be representative of the broader population of family caregivers of Korean LTCF residents, among whom spouses and older, less digitally connected caregivers are probably underrepresented. The online recruitment approach therefore likely introduced selection bias rather than merely raising its possibility, and may have altered the distribution of K-rPATD scores relative to the broader caregiver population; the direction and magnitude of this bias cannot be determined from the present data. Estimates for the spouse category were correspondingly imprecise, as only 18 respondents were spouses, which is reflected in their wide confidence intervals. Third, the self-reported nature of the data collected through a single online survey may have introduced recall bias and common method bias, with the latter potentially inflating the observed associations between the self-reported independent and dependent variables. Fourth, the generalizability of these findings may be limited to the Korean long-term care context, and cross-cultural replication is warranted. Fifth, the explanatory power of the regression models was relatively limited (adjusted R² range: 0.015–0.081), particularly for the involvement, willingness to stop medications, and satisfaction domains. This suggests that caregivers’ attitudes in these domains are likely influenced by broader psychosocial factors, such as trust in healthcare providers, emotional investment in caregiving, and interpersonal communication, which were not captured in the present study. Sixth, experience of ADEs was assessed by caregiver report and categorized as “yes” versus “no or unknown.” The proportion of caregivers who responded “unknown” (19.0%) was substantial. Grouping “unknown” with “no” facilitated binary analysis but may have resulted in exposure misclassification, particularly if some residents in the “unknown” group had experienced ADEs. Seventh, monthly medication costs were treated as ordinal variables with five tiers, and a linear trend across the tiers was assumed in the regression models. Given that the monetary intervals between tiers were unequal, this assumption may not fully capture the true dose-response relationship between medication costs and caregivers’ attitudes toward deprescribing. The survey also recorded the amount spent on the resident’s medicines without identifying who paid it, so the extent to which this expenditure represented a direct financial burden on the caregiver is unknown. Eighth, the number of medications was reported by caregivers, who might have considered fixed-dose combination products as a single medication. This may partly explain why the mean number of medications observed in this study (5.6) was lower than the figure of 10.6 previously reported for Korean LTCF residents [7]. This discrepancy also likely reflects differences in measurement between medication counts obtained from prescribing records and those reported by caregivers, who may be unaware of medications they do not routinely handle, such as as-needed or externally prescribed drugs. The relatively low mean number of chronic conditions reported (2.2) is consistent with this caregiver-report limitation and may underestimate the residents’ true comorbidity burden. The possibility of underestimation should be considered when interpreting the associated findings. Ninth, a large number of statistical tests were performed (16 measured predictors represented by 18 regression terms across six regression models). To address this, we applied a Benjamini–Hochberg false discovery rate (FDR) correction and report unadjusted and FDR-adjusted p values for all coefficients (Supplementary Table S1); associations that did not survive this correction, particularly those observed for caregiver and resident sociodemographic characteristics, are regarded as exploratory, hypothesis-generating findings that require replication. Tenth, several predictors overlap conceptually with individual K-rPATD items: monthly medication cost corresponds closely to the burden item on medicines being expensive, the number of medications to the burden items on taking many or too many medicines, and ADE experience to the appropriateness item on medicines causing side effects. This content overlap may have contributed to the strength of the corresponding associations, which should therefore be read as convergent rather than as evidence of independent determinants. Eleventh, internal consistency was low for the concerns about stopping subscale (Cronbach’s alpha 0.563) and modest for involvement (0.637) in the validation study [25]; findings for these two domains, including the association between ADE experience and concerns about stopping, should be interpreted with corresponding caution. This study was conducted and reported in accordance with the STROBE checklist for cross-sectional studies, and its findings are interpreted as associational and hypothesis-generating.

Despite these limitations, this study has several strengths. To our knowledge, this is one of the few studies to examine a broad range of caregiver-, resident-, and medication-related factors associated with caregivers’ attitudes toward deprescribing specifically in an LTCF setting, moving beyond the willingness-focused approach of previous studies. Based on a large nationwide online-panel sample of 973 caregivers and using the validated K-rPATD, the findings provide a multidimensional understanding of the factors associated with caregivers’ attitudes and offer practical implications for developing deprescribing strategies tailored to the long-term care context. Additional strengths include the simultaneous assessment of six distinct attitudinal domains rather than willingness alone, the quantification of structural and economic factors—such as the prescribing pathway and medication cost—that have rarely been examined in previous attitudinal studies, and the methodological rigor afforded by false discovery rate correction, heteroscedasticity-consistent standard errors, and sensitivity analyses examining alternative codings of key exposures.

## Conclusions

This study identified several correlates of family caregivers’ attitudes toward deprescribing in South Korean LTCFs. After correction for multiple comparisons, the associations that remained were caregiver-reported ADE experience with greater perceived burden, lower perceived appropriateness of current medicines, and greater concerns about stopping; higher monthly medication cost with greater burden and concerns about stopping; a greater number of medications with greater burden; and a greater number of chronic conditions with greater concerns about stopping and involvement. Caregivers’ attitudes were therefore most consistently linked to tangible medication-related experience rather than to sociodemographic characteristics, although the models explained only a small proportion of outcome variability.

These findings suggest that efforts to promote deprescribing in LTCFs may be most relevant to caregivers for whom the burden of medicines is already salient — those reporting adverse drug events, higher medication costs, or more complex regimens. As a secondary, exploratory observation that did not survive correction for multiple comparisons (FDR-adjusted *p* = 0.186), dual-track prescribing was associated with greater concerns about stopping medications. Whether a fragmented prescribing structure contributes to caregivers’ uncertainty about responsibility for medication decisions, and whether integrated prescribing information, multidisciplinary medication review, or a clearly identified clinician responsible for medication coordination would help, requires dedicated study.

## Supplementary Information

Supplementary Material 1: Supplementary Tables S1–S3. Table S1, complete regression results for all 108 coefficients, with unadjusted, false discovery rate-adjusted, and heteroscedasticity-consistent (HC3) p values; Table S2, sensitivity analyses (categorical modeling of monthly medication cost with tests for departure from linearity, three-level coding of adverse drug event experience, a complete-case analysis excluding "unknown" responses, and categorical modeling of monthly dietary supplement cost in the satisfaction model); Table S3, ordinal logistic regression for the two single-item outcomes, regression diagnostics, and the model additionally adjusted for primary-caregiver status.

Supplementary Material 2: Completed STROBE checklist for cross-sectional studies.

## Data Availability

The datasets generated and analyzed during the current study are not publicly available because the participants did not consent to public data sharing and the study protocol approved by the Institutional Review Board of Chung-Ang University does not permit public deposition of the dataset. The datasets are available from the corresponding author on reasonable request.

## Abbreviations

ADE: Adverse drug event
CI: Confidence interval
FDR: False discovery rate
HC3: Heteroscedasticity-consistent (type 3) standard errors
KAP: Knowledge-Attitude-Practice
K-rPATD: Korean version of the revised Patients’ Attitudes Towards Deprescribing questionnaire
KRW: South Korean Won
LTCF: Long-term care facility
PIM: Potentially inappropriate medication
rPATD: revised Patients’ Attitudes Towards Deprescribing
STROBE: Strengthening the Reporting of Observational Studies in Epidemiology
USD: US dollar
VIF: Variance inflation factor
