# Supplementary material for: Factors associated with family caregivers’ attitudes toward deprescribing in long-term care facilities: a cross-sectional study in South Korea
Source: BMC Geriatr. 2026 Oct 1;26:1276. doi: 10.1186/s12877-026-08333-6 (PMC13651151; doi:10.1186/s12877-026-08333-6)
Supplement: Supplementary file 2 [file 12877_2026_8333_MOESM2_ESM.docx]

**Supplementary Material 1. Supplementary Tables S1–S3**

Factors Associated with Family Caregivers' Attitudes Toward Deprescribing in Long-Term Care Facilities: A Cross-Sectional Study in South Korea

All analyses are based on the 973 family caregivers described in the main text. In the tables below, "caregiver" denotes the family caregiver and "resident" the long-term care facility resident cared for.

**Supplementary Table S1. Unadjusted, false discovery rate (FDR)-adjusted, and robust (HC3) p values for all 108 regression coefficients (N = 973).**

| **Outcome domain** | **Independent variable** | **B** | **95% CI** | **p value** | **FDR-adjusted p** | **Robust (HC3) p** |
| --- | --- | --- | --- | --- | --- | --- |
| Burden | Caregiver sex (female vs. male) | −0.005 | (−0.098, 0.089) | 0.920 | 0.979 | 0.921 |
| Burden | Caregiver age group (per 1-category increase) | 0.013 | (−0.047, 0.074) | 0.662 | 0.874 | 0.663 |
| Burden | Caregiver education level (per 1-level increase) | 0.022 | (−0.109, 0.154) | 0.739 | 0.902 | 0.760 |
| Burden | Monthly household income (per 1-category increase) | 0.021 | (−0.020, 0.062) | 0.317 | 0.618 | 0.346 |
| Burden | Relationship: grandchild (vs. child) | −0.008 | (−0.177, 0.161) | 0.925 | 0.979 | 0.928 |
| Burden | Relationship: spouse (vs. child) | −0.276 | (−0.632, 0.081) | 0.129 | 0.511 | 0.189 |
| Burden | Relationship: other (vs. child) | 0.029 | (−0.111, 0.168) | 0.689 | 0.897 | 0.683 |
| Burden | Resident sex (female vs. male) | −0.024 | (−0.120, 0.072) | 0.620 | 0.874 | 0.616 |
| Burden | Resident age group (per 1-category increase) | 0.006 | (−0.063, 0.074) | 0.872 | 0.971 | 0.874 |
| Burden | Duration of stay (per 1-category increase) | 0.005 | (−0.045, 0.056) | 0.832 | 0.936 | 0.827 |
| Burden | Long-term care grade (per 1-grade increase) | 0.009 | (−0.027, 0.045) | 0.639 | 0.874 | 0.634 |
| Burden | Dementia (yes vs. no) | 0.010 | (−0.082, 0.103) | 0.825 | 0.936 | 0.826 |
| Burden | Number of chronic conditions (per 1 increase) | 0.012 | (−0.014, 0.039) | 0.372 | 0.678 | 0.357 |
| Burden | Number of medications (per 1 increase) | 0.026 | (0.013, 0.039) | <0.001 | 0.007 | <0.001 |
| Burden | Experience of adverse drug events (yes vs. no/unknown) | 0.206 | (0.080, 0.333) | 0.001 | 0.019 | 0.001 |
| Burden | Dual-track prescribing (vs. facility-only) | 0.064 | (−0.042, 0.170) | 0.237 | 0.592 | 0.267 |
| Burden | Monthly medication cost (per 1-tier increase) | 0.085 | (0.038, 0.133) | <0.001 | 0.015 | <0.001 |
| Burden | Monthly dietary supplement cost (per 1-tier increase) | −0.008 | (−0.052, 0.036) | 0.734 | 0.902 | 0.736 |
| Appropriateness | Caregiver sex (female vs. male) | −0.047 | (−0.136, 0.041) | 0.295 | 0.617 | 0.292 |
| Appropriateness | Caregiver age group (per 1-category increase) | −0.014 | (−0.071, 0.044) | 0.640 | 0.874 | 0.634 |
| Appropriateness | Caregiver education level (per 1-level increase) | 0.001 | (−0.123, 0.126) | 0.982 | 0.998 | 0.983 |
| Appropriateness | Monthly household income (per 1-category increase) | −0.023 | (−0.062, 0.015) | 0.236 | 0.592 | 0.265 |
| Appropriateness | Relationship: grandchild (vs. child) | 0.026 | (−0.133, 0.186) | 0.745 | 0.902 | 0.764 |
| Appropriateness | Relationship: spouse (vs. child) | 0.010 | (−0.327, 0.347) | 0.953 | 0.987 | 0.960 |
| Appropriateness | Relationship: other (vs. child) | −0.055 | (−0.187, 0.077) | 0.415 | 0.690 | 0.396 |
| Appropriateness | Resident sex (female vs. male) | 0.046 | (−0.045, 0.137) | 0.325 | 0.618 | 0.322 |
| Appropriateness | Resident age group (per 1-category increase) | −0.031 | (−0.095, 0.034) | 0.349 | 0.650 | 0.339 |
| Appropriateness | Duration of stay (per 1-category increase) | 0.001 | (−0.047, 0.049) | 0.959 | 0.987 | 0.961 |
| Appropriateness | Long-term care grade (per 1-grade increase) | 0.000 | (−0.034, 0.034) | 0.998 | 0.998 | 0.998 |
| Appropriateness | Dementia (yes vs. no) | −0.037 | (−0.125, 0.050) | 0.404 | 0.687 | 0.411 |
| Appropriateness | Number of chronic conditions (per 1 increase) | −0.015 | (−0.040, 0.010) | 0.253 | 0.592 | 0.242 |
| Appropriateness | Number of medications (per 1 increase) | −0.015 | (−0.027, −0.002) | 0.023 | 0.186 | 0.019 |
| Appropriateness | Experience of adverse drug events (yes vs. no/unknown) | −0.208 | (−0.327, −0.088) | <0.001 | 0.018 | 0.001 |
| Appropriateness | Dual-track prescribing (vs. facility-only) | −0.079 | (−0.179, 0.021) | 0.122 | 0.507 | 0.133 |
| Appropriateness | Monthly medication cost (per 1-tier increase) | −0.057 | (−0.101, −0.012) | 0.013 | 0.141 | 0.016 |
| Appropriateness | Monthly dietary supplement cost (per 1-tier increase) | −0.021 | (−0.063, 0.021) | 0.322 | 0.618 | 0.322 |
| Concerns about stopping | Caregiver sex (female vs. male) | −0.044 | (−0.126, 0.037) | 0.287 | 0.617 | 0.289 |
| Concerns about stopping | Caregiver age group (per 1-category increase) | 0.010 | (−0.042, 0.063) | 0.703 | 0.902 | 0.712 |
| Concerns about stopping | Caregiver education level (per 1-level increase) | −0.102 | (−0.217, 0.012) | 0.081 | 0.362 | 0.103 |
| Concerns about stopping | Monthly household income (per 1-category increase) | 0.020 | (−0.015, 0.056) | 0.257 | 0.592 | 0.261 |
| Concerns about stopping | Relationship: grandchild (vs. child) | −0.007 | (−0.154, 0.140) | 0.924 | 0.979 | 0.928 |
| Concerns about stopping | Relationship: spouse (vs. child) | 0.055 | (−0.256, 0.365) | 0.730 | 0.902 | 0.751 |
| Concerns about stopping | Relationship: other (vs. child) | 0.078 | (−0.044, 0.200) | 0.208 | 0.592 | 0.211 |
| Concerns about stopping | Resident sex (female vs. male) | −0.064 | (−0.148, 0.019) | 0.132 | 0.511 | 0.139 |
| Concerns about stopping | Resident age group (per 1-category increase) | −0.014 | (−0.073, 0.046) | 0.647 | 0.874 | 0.669 |
| Concerns about stopping | Duration of stay (per 1-category increase) | 0.011 | (−0.033, 0.055) | 0.624 | 0.874 | 0.627 |
| Concerns about stopping | Long-term care grade (per 1-grade increase) | −0.022 | (−0.054, 0.009) | 0.166 | 0.577 | 0.181 |
| Concerns about stopping | Dementia (yes vs. no) | −0.004 | (−0.085, 0.076) | 0.915 | 0.979 | 0.915 |
| Concerns about stopping | Number of chronic conditions (per 1 increase) | 0.038 | (0.015, 0.062) | 0.001 | 0.019 | 0.002 |
| Concerns about stopping | Number of medications (per 1 increase) | −0.007 | (−0.018, 0.005) | 0.242 | 0.592 | 0.273 |
| Concerns about stopping | Experience of adverse drug events (yes vs. no/unknown) | 0.255 | (0.146, 0.365) | <0.001 | <0.001 | <0.001 |
| Concerns about stopping | Dual-track prescribing (vs. facility-only) | 0.108 | (0.016, 0.200) | 0.022 | 0.186 | 0.020 |
| Concerns about stopping | Monthly medication cost (per 1-tier increase) | 0.067 | (0.026, 0.109) | 0.001 | 0.019 | 0.002 |
| Concerns about stopping | Monthly dietary supplement cost (per 1-tier increase) | 0.002 | (−0.037, 0.040) | 0.938 | 0.984 | 0.939 |
| Involvement | Caregiver sex (female vs. male) | −0.047 | (−0.126, 0.033) | 0.252 | 0.592 | 0.259 |
| Involvement | Caregiver age group (per 1-category increase) | −0.027 | (−0.079, 0.024) | 0.297 | 0.617 | 0.332 |
| Involvement | Caregiver education level (per 1-level increase) | −0.019 | (−0.131, 0.094) | 0.742 | 0.902 | 0.759 |
| Involvement | Monthly household income (per 1-category increase) | 0.036 | (0.001, 0.071) | 0.043 | 0.260 | 0.052 |
| Involvement | Relationship: grandchild (vs. child) | −0.022 | (−0.166, 0.121) | 0.760 | 0.902 | 0.783 |
| Involvement | Relationship: spouse (vs. child) | −0.154 | (−0.458, 0.150) | 0.320 | 0.618 | 0.465 |
| Involvement | Relationship: other (vs. child) | −0.036 | (−0.156, 0.083) | 0.548 | 0.833 | 0.504 |
| Involvement | Resident sex (female vs. male) | 0.013 | (−0.069, 0.095) | 0.755 | 0.902 | 0.761 |
| Involvement | Resident age group (per 1-category increase) | 0.023 | (−0.035, 0.082) | 0.433 | 0.695 | 0.447 |
| Involvement | Duration of stay (per 1-category increase) | −0.011 | (−0.054, 0.033) | 0.629 | 0.874 | 0.626 |
| Involvement | Long-term care grade (per 1-grade increase) | −0.017 | (−0.048, 0.014) | 0.274 | 0.604 | 0.281 |
| Involvement | Dementia (yes vs. no) | −0.031 | (−0.110, 0.048) | 0.437 | 0.695 | 0.440 |
| Involvement | Number of chronic conditions (per 1 increase) | 0.038 | (0.015, 0.060) | 0.001 | 0.019 | <0.001 |
| Involvement | Number of medications (per 1 increase) | 0.006 | (−0.006, 0.017) | 0.326 | 0.618 | 0.327 |
| Involvement | Experience of adverse drug events (yes vs. no/unknown) | 0.068 | (−0.039, 0.176) | 0.212 | 0.592 | 0.242 |
| Involvement | Dual-track prescribing (vs. facility-only) | 0.052 | (−0.039, 0.142) | 0.263 | 0.592 | 0.250 |
| Involvement | Monthly medication cost (per 1-tier increase) | 0.026 | (−0.014, 0.066) | 0.208 | 0.592 | 0.216 |
| Involvement | Monthly dietary supplement cost (per 1-tier increase) | 0.003 | (−0.035, 0.040) | 0.888 | 0.978 | 0.887 |
| Willingness to stop medications | Caregiver sex (female vs. male) | −0.074 | (−0.189, 0.041) | 0.207 | 0.592 | 0.207 |
| Willingness to stop medications | Caregiver age group (per 1-category increase) | −0.032 | (−0.106, 0.042) | 0.401 | 0.687 | 0.454 |
| Willingness to stop medications | Caregiver education level (per 1-level increase) | 0.037 | (−0.125, 0.199) | 0.655 | 0.874 | 0.673 |
| Willingness to stop medications | Monthly household income (per 1-category increase) | 0.046 | (−0.004, 0.096) | 0.070 | 0.346 | 0.088 |
| Willingness to stop medications | Relationship: grandchild (vs. child) | −0.062 | (−0.269, 0.145) | 0.555 | 0.833 | 0.595 |
| Willingness to stop medications | Relationship: spouse (vs. child) | −0.154 | (−0.591, 0.284) | 0.491 | 0.768 | 0.514 |
| Willingness to stop medications | Relationship: other (vs. child) | −0.074 | (−0.246, 0.098) | 0.399 | 0.687 | 0.403 |
| Willingness to stop medications | Resident sex (female vs. male) | 0.103 | (−0.015, 0.221) | 0.087 | 0.378 | 0.084 |
| Willingness to stop medications | Resident age group (per 1-category increase) | 0.102 | (0.018, 0.185) | 0.018 | 0.172 | 0.024 |
| Willingness to stop medications | Duration of stay (per 1-category increase) | −0.039 | (−0.101, 0.023) | 0.221 | 0.592 | 0.242 |
| Willingness to stop medications | Long-term care grade (per 1-grade increase) | 0.010 | (−0.034, 0.054) | 0.664 | 0.874 | 0.659 |
| Willingness to stop medications | Dementia (yes vs. no) | −0.014 | (−0.128, 0.100) | 0.813 | 0.934 | 0.811 |
| Willingness to stop medications | Number of chronic conditions (per 1 increase) | 0.030 | (−0.003, 0.063) | 0.071 | 0.346 | 0.098 |
| Willingness to stop medications | Number of medications (per 1 increase) | 0.012 | (−0.004, 0.028) | 0.149 | 0.535 | 0.176 |
| Willingness to stop medications | Experience of adverse drug events (yes vs. no/unknown) | 0.166 | (0.011, 0.321) | 0.035 | 0.256 | 0.034 |
| Willingness to stop medications | Dual-track prescribing (vs. facility-only) | 0.085 | (−0.045, 0.215) | 0.198 | 0.592 | 0.244 |
| Willingness to stop medications | Monthly medication cost (per 1-tier increase) | 0.055 | (−0.003, 0.113) | 0.063 | 0.343 | 0.060 |
| Willingness to stop medications | Monthly dietary supplement cost (per 1-tier increase) | −0.017 | (−0.071, 0.037) | 0.546 | 0.833 | 0.560 |
| Satisfaction | Caregiver sex (female vs. male) | −0.110 | (−0.217, −0.003) | 0.044 | 0.260 | 0.048 |
| Satisfaction | Caregiver age group (per 1-category increase) | −0.020 | (−0.089, 0.049) | 0.575 | 0.851 | 0.587 |
| Satisfaction | Caregiver education level (per 1-level increase) | −0.154 | (−0.305, −0.003) | 0.046 | 0.260 | 0.073 |
| Satisfaction | Monthly household income (per 1-category increase) | 0.048 | (0.002, 0.095) | 0.042 | 0.260 | 0.045 |
| Satisfaction | Relationship: grandchild (vs. child) | 0.026 | (−0.167, 0.219) | 0.788 | 0.926 | 0.801 |
| Satisfaction | Relationship: spouse (vs. child) | −0.264 | (−0.671, 0.144) | 0.205 | 0.592 | 0.208 |
| Satisfaction | Relationship: other (vs. child) | −0.227 | (−0.387, −0.067) | 0.005 | 0.066 | 0.008 |
| Satisfaction | Resident sex (female vs. male) | 0.013 | (−0.097, 0.123) | 0.813 | 0.934 | 0.817 |
| Satisfaction | Resident age group (per 1-category increase) | −0.045 | (−0.123, 0.033) | 0.262 | 0.592 | 0.263 |
| Satisfaction | Duration of stay (per 1-category increase) | 0.000 | (−0.058, 0.058) | 0.997 | 0.998 | 0.997 |
| Satisfaction | Long-term care grade (per 1-grade increase) | −0.028 | (−0.069, 0.014) | 0.189 | 0.592 | 0.179 |
| Satisfaction | Dementia (yes vs. no) | −0.067 | (−0.173, 0.039) | 0.215 | 0.592 | 0.221 |
| Satisfaction | Number of chronic conditions (per 1 increase) | 0.035 | (0.005, 0.065) | 0.024 | 0.186 | 0.020 |
| Satisfaction | Number of medications (per 1 increase) | −0.006 | (−0.021, 0.009) | 0.438 | 0.695 | 0.457 |
| Satisfaction | Experience of adverse drug events (yes vs. no/unknown) | −0.061 | (−0.205, 0.083) | 0.407 | 0.687 | 0.464 |
| Satisfaction | Dual-track prescribing (vs. facility-only) | −0.055 | (−0.176, 0.067) | 0.377 | 0.678 | 0.368 |
| Satisfaction | Monthly medication cost (per 1-tier increase) | −0.048 | (−0.103, 0.006) | 0.079 | 0.362 | 0.087 |
| Satisfaction | Monthly dietary supplement cost (per 1-tier increase) | 0.038 | (−0.013, 0.088) | 0.141 | 0.525 | 0.143 |

*All 18 regression terms (16 measured predictors; relationship to the resident contributes three dummy variables) were entered simultaneously and mutually adjusted in each of the six models. FDR adjustment was applied across all 108 coefficients using the Benjamini–Hochberg procedure. Robust p values were obtained from heteroscedasticity-consistent (HC3) standard errors. The appropriateness factor is reverse-scored, so that a positive coefficient indicates a stronger belief that the resident's current medicines are appropriate. Associations with an FDR-adjusted p ≥ 0.05 are interpreted as exploratory. B, unstandardized coefficient; CI, confidence interval.*

**Supplementary Table S2. Sensitivity analyses for the specification of the ordinal cost variables and the coding of adverse drug event (ADE) experience.**

**(A) Monthly medication cost modelled as an unordered categorical variable (reference: lowest tier), all six domains (N = 973)**

| **K-rPATD domain** | **Tier 2 vs 1** | **Tier 3 vs 1** | **Tier 4 vs 1** | **Tier 5 vs 1** | **Linear B** | **Departure from linearity** |
| --- | --- | --- | --- | --- | --- | --- |
| Burden | 0.194 (0.001, 0.388) | 0.198 (0.003, 0.394) | 0.339 (0.129, 0.548) | 0.401 (0.166, 0.636) | 0.085 | F(3, 951) = 0.83, p = 0.476 |
| Appropriateness | -0.122 (-0.305, 0.062) | -0.129 (-0.314, 0.056) | -0.209 (-0.408, -0.011) | -0.271 (-0.493, -0.048) | -0.057 | F(3, 951) = 0.31, p = 0.815 |
| Concerns about stopping | 0.147 (-0.022, 0.316) | 0.191 (0.021, 0.361) | 0.298 (0.116, 0.481) | 0.288 (0.083, 0.493) | 0.067 | F(3, 951) = 0.76, p = 0.517 |
| Involvement | -0.002 (-0.167, 0.163) | 0.006 (-0.161, 0.173) | 0.003 (-0.175, 0.182) | 0.127 (-0.073, 0.328) | 0.026 | F(3, 951) = 0.86, p = 0.459 |
| Willingness to stop medications | -0.011 (-0.249, 0.227) | 0.008 (-0.232, 0.248) | 0.120 (-0.137, 0.377) | 0.170 (-0.119, 0.459) | 0.055 | F(3, 951) = 0.36, p = 0.782 |
| Satisfaction | -0.020 (-0.242, 0.202) | -0.074 (-0.298, 0.150) | -0.101 (-0.341, 0.138) | -0.195 (-0.464, 0.075) | -0.048 | F(3, 951) = 0.11, p = 0.954 |

*Values are B (95% CI). “Linear B” is the coefficient per one-tier increase from the main model. The F test compares the categorical specification with the linear specification; p ≥ 0.05 indicates no detectable departure from linearity.*

**(B) ADE experience modelled as a three-level variable (reference: no) (N = 973)**

| **K-rPATD domain** | **ADE = yes: B (95% CI)** | **p** | **ADE = unknown: B (95% CI)** | **p** |
| --- | --- | --- | --- | --- |
| Burden | 0.226 (0.097, 0.354) | <0.001 | 0.086 (-0.031, 0.203) | 0.148 |
| Appropriateness | -0.232 (-0.354, -0.110) | <0.001 | -0.108 (-0.219, 0.002) | 0.054 |
| Concerns about stopping | 0.254 (0.142, 0.367) | <0.001 | -0.004 (-0.106, 0.097) | 0.932 |
| Involvement | 0.052 (-0.057, 0.162) | 0.349 | -0.072 (-0.171, 0.028) | 0.158 |
| Willingness to stop medications | 0.160 (0.001, 0.318) | 0.048 | -0.029 (-0.172, 0.114) | 0.692 |
| Satisfaction | -0.087 (-0.234, 0.061) | 0.248 | -0.115 (-0.249, 0.018) | 0.091 |

**(C) Complete-case analysis excluding respondents with unknown ADE status (N = 788)**

| **K-rPATD domain** | **ADE (yes vs. no): B (95% CI)** | **p** |
| --- | --- | --- |
| Burden | 0.218 (0.089, 0.347) | <0.001 |
| Appropriateness | -0.228 (-0.351, -0.104) | <0.001 |
| Concerns about stopping | 0.249 (0.134, 0.363) | <0.001 |
| Involvement | 0.041 (-0.069, 0.152) | 0.461 |
| Willingness to stop medications | 0.146 (-0.012, 0.304) | 0.069 |
| Satisfaction | -0.111 (-0.262, 0.040) | 0.150 |

In panel B the “unknown” category did not differ significantly from “no” in any domain. In panel C the estimates for ADE = yes were similar to those of the main analysis for burden, appropriateness and concerns about stopping, and these associations remained significant; the weaker association with willingness to stop medications was attenuated and no longer nominally significant (B = 0.146, p = 0.069). Together these support the yes-versus-no/unknown coding used in the main models for the principal findings, while indicating that the willingness association is sensitive to the treatment of “unknown” responses. All models were adjusted for the full set of covariates.

**(D) Monthly dietary supplement cost modelled as an unordered categorical variable in the satisfaction model (reference: lowest tier) (N = 973)**

| **Dietary supplement cost tier** | **B (95% CI)** | **p** |
| --- | --- | --- |
| Tier 2 vs. tier 1 | 0.146 (-0.013, 0.304) | 0.071 |
| Tier 3 vs. tier 1 | 0.224 (0.067, 0.382) | 0.005 |
| Tier 4 vs. tier 1 | 0.060 (-0.123, 0.244) | 0.519 |
| Tier 5 vs. tier 1 | 0.245 (0.009, 0.480) | 0.041 |
| **Joint test for the categorical term** | F(4, 951) = 2.74 | 0.027 |

A departure from linearity was detected for dietary supplement cost in the satisfaction model (F(3, 951) = 2.93, p = 0.033). Modelled categorically, the term was jointly associated with satisfaction in a non-monotonic pattern, but the association was nominal and would not survive FDR adjustment. The remaining coefficients were essentially unchanged (for example, number of chronic conditions 0.035 → 0.033; caregiver education −0.154 → −0.156), so the linear specification was retained in the main models to keep all six models identically specified.

**Supplementary Table S3. Further sensitivity analyses.**

**(A) Ordinal logistic regression for the two single-item global outcomes, compared with the linear models (N = 973)**

| **Outcome** | **Independent variable** | **Ordinal OR (95% CI)** | **p** | **Linear B** | **p** | **Same direction** |
| --- | --- | --- | --- | --- | --- | --- |
| Willingness to stop medications | Caregiver sex (female vs. male) | 0.832 (0.648, 1.067) | 0.147 | −0.074 | 0.207 | Yes |
| Willingness to stop medications | Caregiver age group (per 1-category increase) | 0.946 (0.801, 1.116) | 0.508 | −0.032 | 0.401 | Yes |
| Willingness to stop medications | Caregiver education level (per 1-level increase) | 1.095 (0.771, 1.556) | 0.612 | 0.037 | 0.655 | Yes |
| Willingness to stop medications | Monthly household income (per 1-category increase) | 1.102 (0.988, 1.231) | 0.082 | 0.046 | 0.070 | Yes |
| Willingness to stop medications | Relationship: grandchild (vs. child) | 0.917 (0.579, 1.453) | 0.712 | −0.062 | 0.555 | Yes |
| Willingness to stop medications | Relationship: spouse (vs. child) | 0.621 (0.243, 1.582) | 0.318 | −0.154 | 0.491 | Yes |
| Willingness to stop medications | Relationship: other (vs. child) | 0.829 (0.572, 1.200) | 0.320 | −0.074 | 0.399 | Yes |
| Willingness to stop medications | Resident sex (female vs. male) | 1.208 (0.936, 1.559) | 0.146 | 0.103 | 0.087 | Yes |
| Willingness to stop medications | Resident age group (per 1-category increase) | 1.207 (1.005, 1.449) | 0.044 | 0.102 | 0.018 | Yes |
| Willingness to stop medications | Duration of stay (per 1-category increase) | 0.934 (0.816, 1.070) | 0.325 | −0.039 | 0.221 | Yes |
| Willingness to stop medications | Long-term care grade (per 1-grade increase) | 1.014 (0.922, 1.116) | 0.776 | 0.010 | 0.664 | Yes |
| Willingness to stop medications | Dementia (yes vs. no) | 1.003 (0.784, 1.283) | 0.979 | −0.014 | 0.813 | No |
| Willingness to stop medications | Number of chronic conditions (per 1 increase) | 1.084 (1.007, 1.167) | 0.032 | 0.030 | 0.071 | Yes |
| Willingness to stop medications | Number of medications (per 1 increase) | 1.033 (0.996, 1.072) | 0.078 | 0.012 | 0.149 | Yes |
| Willingness to stop medications | Experience of adverse drug events (yes vs. no/unknown) | 1.540 (1.096, 2.164) | 0.013 | 0.166 | 0.035 | Yes |
| Willingness to stop medications | Dual-track prescribing (vs. facility-only) | 1.128 (0.849, 1.499) | 0.405 | 0.085 | 0.198 | Yes |
| Willingness to stop medications | Monthly medication cost (per 1-tier increase) | 1.120 (0.987, 1.270) | 0.079 | 0.055 | 0.063 | Yes |
| Willingness to stop medications | Monthly dietary supplement cost (per 1-tier increase) | 0.962 (0.855, 1.083) | 0.525 | −0.017 | 0.546 | Yes |
| Satisfaction | Caregiver sex (female vs. male) | 0.771 (0.599, 0.992) | 0.043 | −0.110 | 0.044 | Yes |
| Satisfaction | Caregiver age group (per 1-category increase) | 0.961 (0.817, 1.131) | 0.633 | −0.020 | 0.575 | Yes |
| Satisfaction | Caregiver education level (per 1-level increase) | 0.669 (0.466, 0.961) | 0.030 | −0.154 | 0.046 | Yes |
| Satisfaction | Monthly household income (per 1-category increase) | 1.130 (1.012, 1.262) | 0.030 | 0.048 | 0.042 | Yes |
| Satisfaction | Relationship: grandchild (vs. child) | 1.054 (0.667, 1.664) | 0.823 | 0.026 | 0.788 | Yes |
| Satisfaction | Relationship: spouse (vs. child) | 0.543 (0.207, 1.424) | 0.214 | −0.264 | 0.205 | Yes |
| Satisfaction | Relationship: other (vs. child) | 0.594 (0.404, 0.872) | 0.008 | −0.227 | 0.005 | Yes |
| Satisfaction | Resident sex (female vs. male) | 1.047 (0.809, 1.356) | 0.725 | 0.013 | 0.813 | Yes |
| Satisfaction | Resident age group (per 1-category increase) | 0.918 (0.765, 1.102) | 0.357 | −0.045 | 0.262 | Yes |
| Satisfaction | Duration of stay (per 1-category increase) | 0.976 (0.852, 1.119) | 0.730 | 0.000 | 0.997 | Yes |
| Satisfaction | Long-term care grade (per 1-grade increase) | 0.940 (0.854, 1.034) | 0.202 | −0.028 | 0.189 | Yes |
| Satisfaction | Dementia (yes vs. no) | 0.873 (0.680, 1.120) | 0.285 | −0.067 | 0.215 | Yes |
| Satisfaction | Number of chronic conditions (per 1 increase) | 1.090 (1.015, 1.169) | 0.017 | 0.035 | 0.024 | Yes |
| Satisfaction | Number of medications (per 1 increase) | 0.982 (0.947, 1.018) | 0.318 | −0.006 | 0.438 | Yes |
| Satisfaction | Experience of adverse drug events (yes vs. no/unknown) | 0.862 (0.609, 1.221) | 0.404 | −0.061 | 0.407 | Yes |
| Satisfaction | Dual-track prescribing (vs. facility-only) | 0.891 (0.671, 1.183) | 0.424 | −0.055 | 0.377 | Yes |
| Satisfaction | Monthly medication cost (per 1-tier increase) | 0.916 (0.806, 1.041) | 0.178 | −0.048 | 0.079 | Yes |
| Satisfaction | Monthly dietary supplement cost (per 1-tier increase) | 1.077 (0.957, 1.212) | 0.220 | 0.038 | 0.141 | Yes |

Willingness to stop medications and satisfaction are single 5-point Likert items; they were therefore refitted using ordinal logistic regression. The direction of association agreed with the linear model for 35 of the 36 coefficients; the single exception (dementia in the willingness model) had an odds ratio of 1.003 and was indistinguishable from the null. Nominal significance differed for the number of chronic conditions in the willingness model (linear p = 0.071; ordinal p = 0.032), but neither estimate survives FDR adjustment and no principal conclusion changed. OR, odds ratio.

**(B) Regression diagnostics for the six main models (N = 973)**

| **K-rPATD domain** | **Breusch–Pagan p** | **Maximum Cook's distance** | **Observations with Cook's distance > 1** | **Adjusted R²** |
| --- | --- | --- | --- | --- |
| Burden | 0.299 | 0.024 | 0 | 0.055 |
| Appropriateness | 0.032 | 0.023 | 0 | 0.046 |
| Concerns about stopping | 0.704 | 0.022 | 0 | 0.081 |
| Involvement | 0.161 | 0.029 | 0 | 0.026 |
| Willingness to stop medications | 0.068 | 0.022 | 0 | 0.027 |
| Satisfaction | 0.281 | 0.018 | 0 | 0.015 |

*All variance inflation factors were below 2.1 (maximum 2.02, mean 1.29). Homoscedasticity was satisfied in five of the six models. No observation exceeded the conventional Cook's distance threshold of 1.*

**(C) Primary-caregiver status added as a covariate: all six models (N = 973)**

| **K-rPATD domain** | **Primary caregiver (yes vs. no): B (95% CI)** | **p** |
| --- | --- | --- |
| Burden | -0.039 (-0.177, 0.099) | 0.583 |
| Appropriateness | 0.006 (-0.124, 0.137) | 0.924 |
| Concerns about stopping | 0.137 (0.017, 0.257) | 0.025 |
| Involvement | -0.045 (-0.162, 0.073) | 0.456 |
| Willingness to stop medications | -0.036 (-0.205, 0.133) | 0.677 |
| Satisfaction | 0.018 (-0.140, 0.176) | 0.821 |
| **Largest change in any other coefficient** | 0.033 (concerns about stopping model) | — |

Each of the six main models was refitted with primary-caregiver status added to the full set of covariates. The coefficient for primary-caregiver status was nominally significant only in the concerns about stopping model and would not survive FDR adjustment; it is therefore exploratory. No other coefficient in any model changed by more than 0.033, and every FDR-surviving association retained its direction and significance.
